# *Escherichia coli* Meningitis after Rotavirus Gastroenteritis in an Infant

**DOI:** 10.1155/2016/1909260

**Published:** 2016-09-21

**Authors:** Gamze Ozgurhan, Oznur Vermezoglu, Didem Ocal Topcu, Adem Karbuz, Aysel Vehapoglu, Bulent Hacihamdioglu

**Affiliations:** ^1^Department of Pediatrics, Süleymaniye Maternity and Children's Training and Research Hospital, Istanbul, Turkey; ^2^Department of Pediatrics, Okmeydanı Training and Research Hospital, Istanbul, Turkey; ^3^Department of Pediatrics, Bezmiâlem Vakıf University Medical School, Istanbul, Turkey

## Abstract

Although rotavirus gastroenteritis is quite common in the pediatric population, secondary bacterial sepsis following rotavirus infection is a rare clinical entity. Gram-negative bacilli are the fifth most common cause of meningitis in infants but this infection rarely occurs after gastroenteritis. Here, we report a 2.5-month-old infant who developed* Escherichia coli* (*E. coli*) meningitis after acute rotavirus gastroenteritis. The 2.5-month-old male infant with fever, vomiting, and watery diarrhea that started 1 day earlier was admitted to the hospital. Rotavirus antigen in stool sample was positive. He was hospitalized, and fever was measured at 39.5°C on the second day. Lumbar puncture was done for suspicion of meningitis, and cerebrospinal fluid (CSF) findings suggested meningitis. Intravenous vancomycin and cefotaxime were started empirically. Since* E. coli* reproduction was seen in blood culture and CSF culture, treatment was continued with cefotaxime. The patient was discharged with minimal midlevel hydrocephalus findings in cranial ultrasonography and magnetic resonance imaging following 21 days of antibiotics treatment. Septicemia development following rotavirus gastroenteritis is an extremely rare clinical condition. It is vital to start prompt antibiotic treatment as soon as the diagnosis of secondary bacterial infection is made because of high mortality and morbidity rates.

## 1. Introduction

Rotavirus targets and infects intestinal villus enterocytes primarily. There have not been many human studies on the rotavirus pathogenesis; therefore, our current understanding is mainly based on animal studies [1]. What has been revealed so far based on these studies is that maldigestion related diarrhea is caused due to 3 main reasons: (i) destruction of enterocytes critical for digestion, (ii) downregulation of enzymes critical for digestion, and (iii) paracellular leakage in tight junctions between enterocytes due to functional changes. The secretory component of rotavirus diarrhea is caused by activation of the enteric nervous system. There are other factors such as changes in intestinal motility which are suggested to have a role in pathogenesis, but there are no credible studies to support their roles yet [2].

Gastroenteritis and dehydration may be the two most well known rotavirus infection complications, but there are others such as seizure, meningoencephalitis, and respiratory illness. Rotavirus has been associated with many systemic illnesses, yet there is no proof that extraintestinal spread of rotavirus has been causing these illnesses since this is difficult to prove as this form of the disease is rare [1–5].

Herein, we report a two-month-old infant who developed* Escherichia coli* (*E. coli*) meningitis after acute rotavirus gastroenteritis.

## 2. Case

The 2.5-month-old male infant with fever, vomiting, and watery diarrhea that started 1 day earlier was admitted to the hospital. Past medical and family history revealed that he was born with term delivery with no special postnatal history. Apart from mild dehydration and irritation, physical examination was normal. Initial leukocyte count was 10,100/mm^3^ and C-reactive protein (CRP) was 19.3 mg/L (references: 0–8). Rotavirus antigen in stool sample was positive. Rotavirus was detected using the Combi-Strip fecal rapid detection system (Coris BioConcept, Belgium). No pathogenic bacteria reproduction was seen in the stool culture.

He was hospitalized because of oral intolerance and dehydration due to rotavirus gastroenteritis. Fever measured on the second day of hospitalization was 39.5°C and he was irritable on physical examination. Lumbar puncture was done for suspicion of meningitides. In analysis of the cerebrospinal fluid (CSF), neutrophilic pleocytosis (800 leukocytes/mm^3^; 68% PMNL, 32% lymphocytes), elevated protein (127 mg/dL), and reduced glucose concentration (11 mg/dL, simultaneous blood glucose: 91 mg/dL) were found. Leukocyte count and CRP at onset of septicemia were elevated to 11,400 cells/mm^3^ and 433 mg/L, respectively.

Intravenous vancomycin and cefotaxime were started empirically. Since* E. coli* reproduction was seen in blood culture and CSF culture, treatment was continued with cefotaxime. The patient was discharged with minimal midlevel hydrocephalus findings in cranial ultrasonography and magnetic resonance imaging following 21 days of antibiotics treatment.

## 3. Discussion

We describe a 2-month-old infant with rotavirus gastroenteritis who developed Gram-negative sepsis and* E. coli* meningitis. There are a small number of septicemia cases following rotavirus gastroenteritis in the literature [6–10], yet there are no cases where meningitis caused by enteric organisms after rotavirus gastroenteritis is reported.

Some studies concluded that secondary bacterial infection development after gastroenteritis was extremely rare. Actually, they explained the fact that secondary infections are not observed more often by not having the sufficient amount of blood culture during rotavirus infection [8]. In our case, during rotavirus gastroenteritis, increased fever was the main reason for us to consider secondary bacterial infection. In addition to gastroenteritis symptoms, other symptoms such as reduced attention to the surroundings, atypical crying, and irritability led us to do a lumbar puncture to exclude central nervous system infection. We applied a 21-day-long antibiotic treatment due to* E. coli* reproduction in the blood and CSF cultures.

As for every bacteremia case, early diagnosis is crucial for postrotavirus gastroenteritis bacteremia. As pointed out by the limited number of cases in the literature, a second peak of the ongoing fever is typical as a sign of bacterial infection development. Other clinical findings vary widely. Leucocytes and acute phase reactants may not increase significantly at the beginning of septicemia [8]. In our case, the initial CRP increased 20 times following bacteremia development. Even though rotavirus infection increases the risk of bacterial infection, secondary bacterial infection caused by the very common rotavirus is extremely rare [11]. This brings to mind the notion that different mechanisms are at play for septicemia development. To gain a better understanding of these mechanisms, studies with larger series are necessary. Having said that, one of the most important ways of protection from these serious complications of rotavirus is by promoting vaccination [12].
